# Determination of Tissue Thermal Conductivity by Measuring and Modeling Temperature Rise Induced in Tissue by Pulsed Focused Ultrasound

**DOI:** 10.1371/journal.pone.0094929

**Published:** 2014-04-17

**Authors:** Tamara Kujawska, Wojciech Secomski, Eleonora Kruglenko, Kazimierz Krawczyk, Andrzej Nowicki

**Affiliations:** Department of Ultrasound, Institute of Fundamental Technological Research, Polish Academy of Sciences,Warsaw, Poland; Michigan State University, United States of America

## Abstract

A tissue thermal conductivity (*K_s_*) is an important parameter which knowledge is essential whenever thermal fields induced in selected organs are predicted. The main objective of this study was to develop an alternative ultrasonic method for determining *K_s_* of tissues *in vitro* suitable for living tissues. First, the method involves measuring of temperature-time *T*(*t*) rises induced in a tested tissue sample by a pulsed focused ultrasound with measured acoustic properties using thermocouples located on the acoustic beam axis. Measurements were performed for 20-cycle tone bursts with a 2 MHz frequency, 0.2 duty-cycle and 3 different initial pressures corresponding to average acoustic powers equal to 0.7 W, 1.4 W and 2.1 W generated from a circular focused transducer with a diameter of 15 mm and f-number of 1.7 in a two-layer system of media: water/beef liver. Measurement results allowed to determine position of maximum heating located inside the beef liver. It was found that this position is at the same axial distance from the source as the maximum peak-peak pressure calculated for each nonlinear beam produced in the two-layer system of media. Then, the method involves modeling of *T*(*t*) at the point of maximum heating and fitting it to the experimental data by adjusting *K_s_*. The averaged value of *K_s_* determined by the proposed method was found to be 0.5±0.02 W/(m·°C) being in good agreement with values determined by other methods. The proposed method is suitable for determining *K_s_* of some animal tissues *in vivo* (for example a rat liver).

## Introduction

The primary goal of many therapeutic applications of focused ultrasound is local heating of selected organs or localized tumors to a desired temperature. A range of beneficial biological effects induced in a targeted lesion by thermal effects depends on exposure level to ultrasound as well as on type of sonicated tissues [1]. When therapy based on the local tissue heating by pulsed focused ultrasound is planned, a selection of acoustic beam parameters providing a desirable temperature rise in treated lesions is crucial. The temperature rise induced in tissues depends not only on the acoustic properties of the beam used but also on the acoustic and thermal properties of tissues intended for heating. Therefore, knowledge of thermal properties of tissues through which the pulsed focused nonlinear ultrasonic waves propagate is necessary for numerical predicting the thermal field induced.

A thermal conductivity is a basic parameter characterizing thermal properties of media. Whenever thermal fields are under consideration it is a major interest to know this parameter. This is especially important for therapeutic applications to ensure safety of any treatment based on the local tissue heating. For example, a local temperature rise by about 4 ∼ 8°C above the physiological norm (37°C) may lead to enhancement of cell protein expression or drug uptake. Ultrasonic regimes are controlled by adjusting a frequency, intensity, pulse duration, duty-cycle and exposure time of generated tone bursts. A typical intensity *I*_SATA_ (spatial-average, temporal-average) of ultrasonic beams used in hyperthermia (for bone healing, physiotherapy, enhancement of drug uptake) is low (0.1 ∼ 2 W/cm^2^), duty-factor is about 0.2, exposure time is long (up to an hour) and a beam width (for −6 dB drop in pressure) in the focal plane may vary between 10% and 20% of the transducer diameter. Very rapid (less than 3 seconds) local heating of tissues to temperatures above 56°C leads to instantaneous cell death. A possibility of a controlled local heating of tissues would allow to control, for example, thermo-ablative necrosis in tumors leaving surrounding tissues intact during treatment using High Intensity Focused Ultrasound (HIFU) technique. A typical intensity in HIFU beam at a point of maximum heating is very high (between several hundred and several thousand W/cm^2^), exposure time is short (up to 3 s), pulse duration may vary between few microsecond and several millisecond, duty-cycle may be between 0.01 and 0.2 and beam width (for −6 dB drop in pressure) may be less than 5% of the transducer diameter due to f-number ≤1. These examples illustrate that the thermal properties of living tissues through which pulsed focused nonlinear acoustic waves propagate should be known for an accurate prognosis of thermal fields induced in them. Values of thermal parameters of several biological tissues *in vitro* are available, for instance, in [2], whereas the thermal properties of tissues *in vivo* are not available.

The main goal of this work was to develop an alternative ultrasonic method suitable for determining thermal conductivity of selected animal tissues *in vivo*. Initially, the proposed method was verified experimentally for a beef liver *in vitro* whose acoustic and thermal properties can be determined experimentally or taken from literature. First, the method involved measuring the temperature-time *T*(*t*) rises induced in a beef liver sample by a pulsed focused nonlinear ultrasonic beam (with measured acoustic properties) using thermocouples located along its acoustic axis. The beam was generated in a two-layer system of parallel media comprising of water and beef liver. Both, the beam focus and maximum heating spots were localized inside the beef liver sample and determined from the temperature-time *T*(*t*) plots registered by thermocouples. It was found that position of the maximum heating is the same for all acoustic power levels used. The two-layer system of media of propagation: water/tissue was selected in order to be able to implement the proposed method for determining *K_s_* of tissues *in vivo* (for example, a rat liver). Focusing pulsed finite-amplitude sound waves inside a liver of an anesthetized rat (after propagation through a layer of water and a thin layer of an abdominal skin) and recording the temperature rise induced during its sonication using a thermocouple located in the beam focus the thermal conductivity of the rat liver *in vivo* can be determined.

Water plays a triple role: as the matching medium, transducer cooling medium (to avoid overheating of water/tissue interface - potentially to avoid skin burns of rats whose livers are intended to be subjected to ultrasound) and nonlinear propagation medium. Absorption in water is small that favors generation of harmonic waves whose absorption nonlinearly increase with frequency and affect on the temperature rise induced in tissues. The path-length that tone bursts propagate through water in the two-layer system of media was determined numerically (using nonlinear propagation model in water) as the axial distance *z*_1_ from the source at which the amplitude of the second harmonic component starts to be discernible.

Both, the nonlinear acoustic pressure and heat sources variations in the pulsed nonlinear beams produced in the two-layer system of media were predicted using our previously developed numerical model based on TAWE approach [3] to numerical solution of the second order nonlinear differential wave equation (transformed KZK equation) for axially symmetric sources. For prediction of temperature fields the commercial software package Abaqus 6.9, being the computer implementation of the Pennes bio-heat transfer equation (for not perfused tissues) solved by the FEM method, was employed. Then, the method involves modeling (for the source boundary condition parameters measured in water) the temperature-time *T*(*t*) rise in the maximum heat spot and fitting the obtained results to the experimental data by adjusting the *K_s_* value of the beef liver. The heat capacity of the beef liver required for the model was taken from the literature. The best correlation between the experimental data and numerical simulation results allowed to determine the thermal conductivity *K_s_* of the beef liver *in vitro*.

## Theoretical Methods

**Figure 1:**
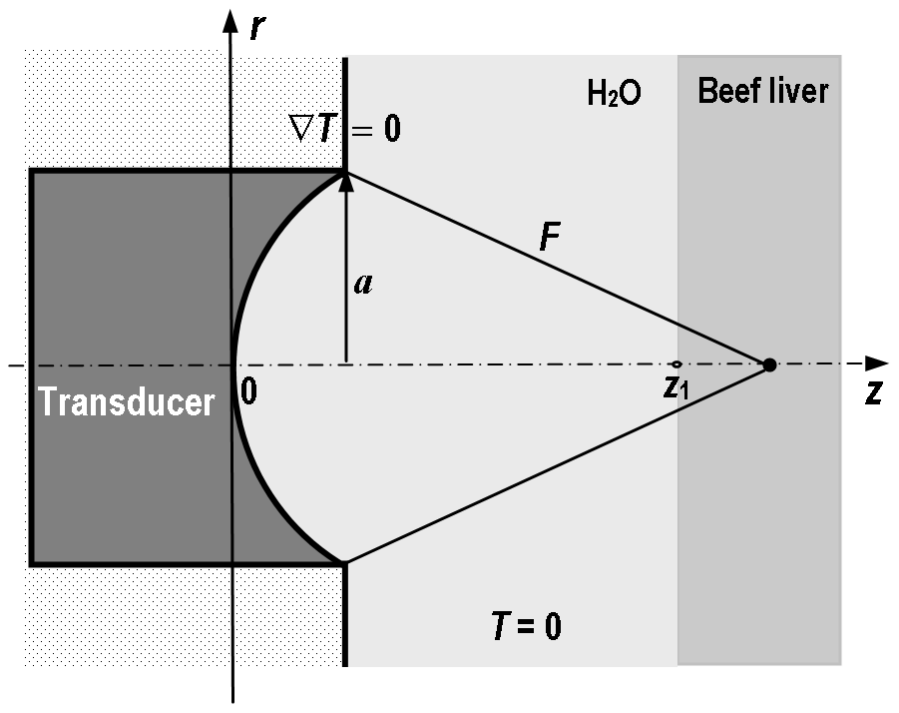
Geometric scheme of a two-layer system of media (water/beef liver) for predicting both, the acoustic pressure and temperature fields induced therein by pulsed focused ultrasonic beams.

It has been assumed that positive direction of a *z* axis overlaps with direction of a finite-amplitude acoustic wave propagation. A circular focused transducer is placed in a cylindrical coordinate system as shown in Fig. 1.

The transducer generates finite-amplitude tone bursts with a given frequency, waveform and PRF in the two-layer system of media (water/tissue). The water/tissue interface is perpendicular to the acoustic beam axis and situated at the axial distance *z*_1_, specific for the transducer used. The distance *z*_1_ was determined theoretically (using nonlinear propagation model in water) as the axial distance from the transducer used at which the amplitude of the second harmonic component starts to be discernible. As shown in previous publications this distance is independent on the source pressure level providing formation of weak or moderate nonlinear beams [4], [5], [6].

In order to predict both, the pressure and heat sources distributions produced in the two-layer system of media by the pulsed focused nonlinear acoustic beam the several boundary condition parameters characterizing both the source and media are required. The transducer parameters being the input data required for the model are: frequency *f*, effective radius *a*_eff_, focal length *F*, source pressure amplitude *P*_0_, radiating aperture apodization function *P*(*r*), initial tone burst waveform *P*(*t*) and duty-cycle (or PRF). These parameters were determined from preliminary measurements in water. The acoustic parameters of media of propagation required for the model are: density *ρ*, sound velocity *c*, frequency-dependent attenuation law *α* (*f*) = *α*_1_ *f ^b^*, and nonlinearity parameter *B/A*.

### Nonlinear Acoustic Pressure and Heat Sources Field Calculation

In order to calculate both the pressure and heat sources variations in the nonlinear beam produced in the two-layer system of media by the pulsed focused ultrasound the original 2D numerical model, recently developed in our laboratory, was used. As was mentioned above, the model is based on the TAWE approach to the numerical solution of the second order nonlinear differential wave equation (transformed KZK equation) for axially symmetric sources. The model accounts for the effects of diffraction, absorption, nonlinear interaction of harmonics as well as for the effects of transmission and reflection at interfaces of parallel media. The model provides a very good simulation of measured acoustic fields generated from planar circular sources (with *ka* >>1) in water when the source pressure level is weak or moderate [7], i.e. when the ratio of the shock formation distance (*l*_D_ = *ρ*_0_ *c*_0_^2^ *λ*_0_/(2+*B/A*)*πP*_0_) to the Rayleigh distance (*R*_0_ = *πa* ^2^/*λ*_0_) is larger than 0.3 (*l*_D_/*R*_0_>0.3). If this condition is true, the model provides a good prediction of measured sound fields produced also by circular focused sources. The comparison of simulated and measured weak or moderate nonlinear sound pressure fields in water produced by circular focused transducers is presented in our previous publications [4], [6]. The numerical model used was an effective research tool capable of quick predicting both, the pressure and heat sources distributions in pulsed nonlinear acoustic beams produced in the two-layer system of media considered by the focused transducer used.

As mentioned above, the model required a number of input data that characterize the boundary conditions of both, the source and media of propagation. The source key parameters included the source pressure amplitude *P*_0_, apodization function *P* (*r*), effective radius *a*_eff_, the initial tone burst pressure-time waveform *P*(*t*) and PRF. These parameters were determined by preliminary measuring of both, the axial and radial pressure variations in the sound beam generated from the transducer used in water and then, by fitting the obtained results to those calculated by adjusting the source parameters.

First, the averaged radial pressure distribution *P* (*r*) was determined from scanning the field laterally in the *xy* plane at the axial distance of 5 mm from the transducer centre using calibrated needle hydrophone. At such short distance the initial wave did not contain any harmonics generated by medium. The use of the needle hydrophone minimized disturbance of the field and the results allowed the source parameters to be determined. The radiating aperture apodization function was initially assumed in the model as *P* (*r*) = *P*_0_ |1−(*r*/*a*_eff_)*^m^* |.

Then, the axial pressure variation *P* (*z*) was measured using the calibrated membrane hydrophone. The radial and axial pressure distributions measured in water were compared with those predicted by our numerical model while the effective radius *a*_eff_, the power exponent *m* and the source pressure amplitude *P*_0_ were varied. The best agreement between the simulated results and measured data allowed to determine the source pressure, effective radius and apodization function.

The pressure-time waveform *P*(*t*) of the initial tone burst used was also determined by the comparison-fitting method and introduced into the model in the form of a polynomial function: *P*(*t*) = [1−|(*t*−*t_c_*)/(*t_b_*−*t_e_*)|*^q^*]·sin[*ω*(*t*−*t_c_*)] for *t_b_*≤*t*≤*t_e_* and *P*(*t*) = 0 for t
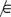
(*t_b_*, *t_e_*), where *t_c_*, *t_b_* and *t_e_* are dimensionless times determining the middle, beginning and end of the initial tone burst, respectively, and *q* is an integer related to its envelope. The pressure-time waveform *P*(*t*) that best reproduced the initial tone burst waveform measured on the acoustic beam axis near to the transducer surface enabled determining the optimal *P*(*t*) waveform, which was used in the model.

The source boundary condition parameters used in the numerical model are presented in Table 1. The acoustic parameters of media of propagation required for the model were taken from the literature [8], [9] and are listed in Table 2.

**Table 1:** The source boundary condition parameters.

Effective diameter 2*a_eff_* [mm]	f-number	Resonance frequency *f*_0_ [MHz]	Source pressure *P*_0_ [MPa]	Pulse duration [µs]	Duty-cycle
13.5	1.7	2	0.263∼0.45	10	0.2

**Table 2:** The acoustic parameters of media of propagation at 25°C temperature.

Medium parameters		Water	Bovine liver
Density, *ρ*_0_	[kg/m^3^]	997	1060
Sound velocity, *c*_0_	[m/s]	1497	1590
Attenuation coefficient, *α*	[Np/(m·Hz*^b^*)]	2.5·10 ^−14^	7.8·10^−6.6^
Nonlinearity parameter, *B/A*		5.2	7
Power index of attenuation dependence on frequency, *b*		2	1.1

### Temperature Field Calculation

For calculation of the temperature rise induced in beef liver sample by pulsed focused ultrasound the Pennes bio-heat transfer equation [10] for not perfused tissues was used:
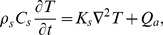
(1)where *ρ_s_*, *C_s_* and *K_s_* are the tissue density, specific heat and thermal conductivity, respectively; *T* is temperature; *t* depicts time;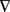 ^2^ denotes the Laplacian. The first term on the right-hand side of Eq. 1 accounts for the effects of heat diffusion and the second one includes the effects of heat sources deposition per unit volume due to absorption of ultrasound energy. According to Kujawska *et al.* [11] the *Q_a_* can be expressed as:
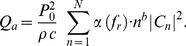
(2)


Here *P*_0_ is the source pressure amplitude, *α* (*f_r_*) = *α*_1_·*f_r_^b^*, where *α*_1_ is the absorption coefficient of the medium of propagation at 1 MHz, *f_r_* is the PRF, *b* is the power index of the absorption dependence on frequency (*b* = 2 for water and *b* = 1∼1.3 for tissues), *C_n_* is the amplitude of the *n*-th spectral component, *n* = *f*/*f_r_* is the number of the spectral component.

Equation (1) was solved numerically for the source used inducing the thermal field in the two-layer system of media: water/beef liver. To solve the problem the thermal insulation boundary conditions were assumed. First, the temperature gradient at the interface source/water was assumed to be equal zero. Next, it was assumed that the acoustic energy is concentrated in a small volume around the beam focus. So, the temperature rise in the region of focus is large while at peripheries, far from the beam axis, is infinitesimal (see Fig. 1). For predicting the temperature fields induced in beef liver by the pulsed focused nonlinear beams the commercial software package Abacus 6.9, being the computer implementation of the above numerical model solved by FEM method, was used. The model required a number of input data that relate to the thermal properties of media of propagation: the specific heat and thermal conductivity. The thermal parameters for water are well studied and were taken from the literature [12]. The value of heat capacity for beef liver *in vitro* was taken from Holmes [2]. The value of thermal conductivity for beef liver was assumed varying between 0.48 W/(m·°C) and 0.52 W/(m·°C) with an increment of 0.01 W/(m·°C) (i. e. within ±4% around the value of 0.5 W/(m·°C), commonly used in publications). The input data of thermal parameters used in the numerical model are listed in Table 3.

**Table 3:** Thermal parameters of the media of propagation.

Thermal parameter		Water	Bovine liver
Thermal conductivity	[W/(m °C)]	4200	3800
Specific heat	[J/(kg °C)]	0.63	0.48∼0.52

## Materials and Methods

### Experimental Setup

**Figure 2:**
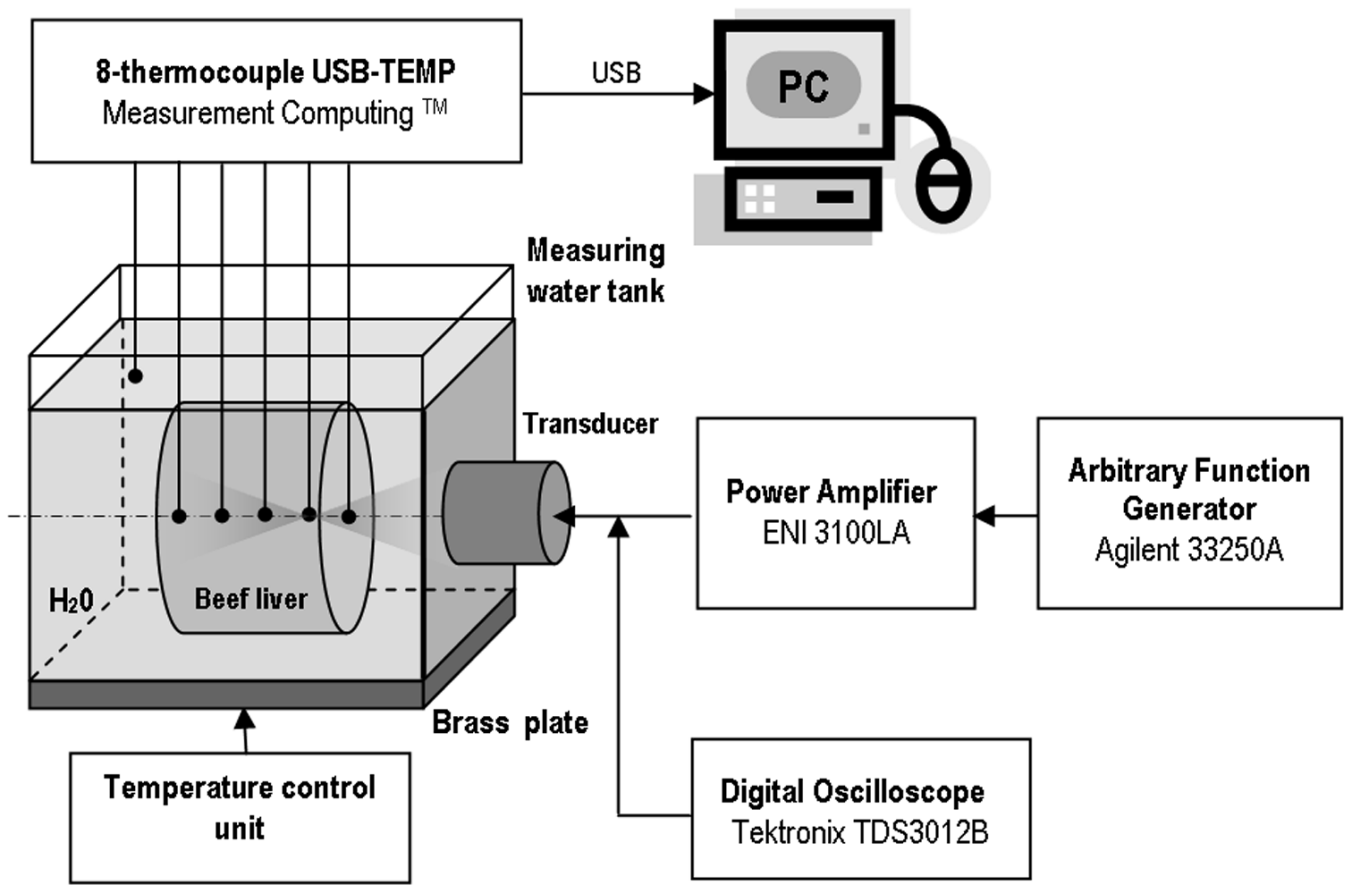
The experimental setup for measuring temperature rises induced in a beef liver sample by pulsed focused ultrasonic beams.

The block-scheme of the experimental setup used for measuring temperature rises induced in beef liver *in vitro* by the pulsed focused nonlinear sound beams used is shown in Fig. 2. All measurements were carried out at a 25°C temperature.

The acoustic pressure tone bursts were generated at the surface of the circular focused transducer (made of Pz26 piezoceramics, Meggitt, Denmark) with a 2 MHz frequency, 15 mm diameter and 25 mm ROC. The transmitting transducer was air-backed, had a quarter-wavelength matching layer and was driven by tone bursts with varied voltage and 0.2 duty-cycle. The transmission electronics were based on an arbitrary function generator (Agilent 33250A, Colorado Springs, USA) that defined the generated tone bursts frequency and pulsing mode. The signal was amplified by a power amplifier (55 dB gain) ENI 3100LA (ENI, Rochester, NY, USA). The output of the amplifier excited the piezoelectric transducer to produce the pulsed focused nonlinear acoustic pressure beam. The voltage applied to the transducer was varied to produce the beam with 3 different acoustic power levels. The average acoustic power was measured using Ultrasonic Power Meter UPM-DT-1E (Ohmic Instruments, Easton, USA) and compared with this resulting from the source pressure measured.

**Figure 3:**
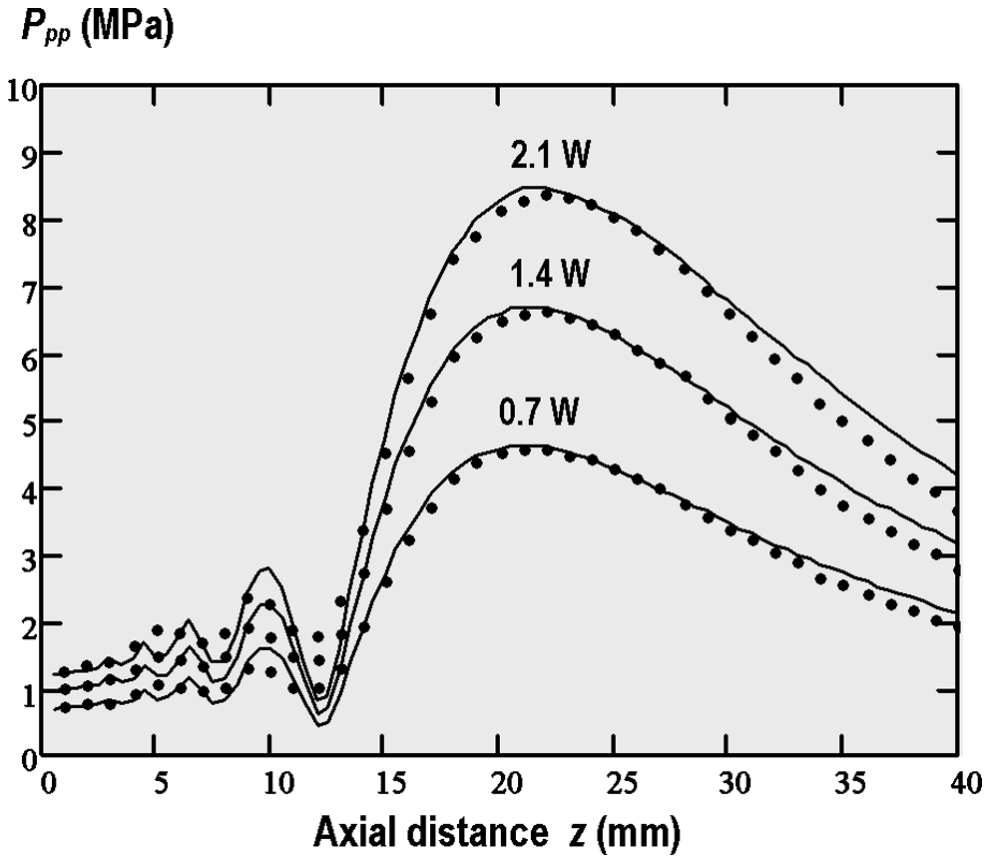
Axial peak-peak pressure variations in the pulsed focused nonlinear acoustic beam produced in water by 20-cycle tone bursts with 0.2 duty-cycle and the average acoustic power of 0.7 W, 1.4 W and 2.1 W. Calculated (solid lines) and measured (points) results.

In order to determine source boundary condition parameters the preliminary measurements in water were carried out. The pressure-time waveforms comprised of 8 cycles were scanned in radial directions in the plane near the transducer surface and recorded by the calibrated 0.2 mm needle hydrophone S/N 1661 (Precision Acoustics, Dorchester, UK). The nonlinearly distorted pressure-time waveforms along and across the acoustic beam axis were recorded by the 0.5 mm PVDF membrane hydrophone with an integrated preamplifier (Unisyn, SN S5-259, Longmont, CO) calibrated up to 40 MHz. The measured axial and radial pressure variations were compared with those simulated numerically. The best agreement between the measurement data and numerical prediction results allowed to determine the source pressure amplitude *P*_0_, radiating aperture effective diameter 2*a*_eff_, apodization function *P*(*r*) and the initial tone burst waveform *P*(*t*). Fig. 3 shows the axial peak-peak (*P_pp_*) pressure variations in the pulsed focused nonlinear sound beams in water calculated and measured for the 8-cycle tone bursts with 3 different source pressure levels corresponding to the weak or moderate nonlinear propagation mode. The obtained results have shown that the best fitting between the experimental data and numerical simulations is provided when the effective diameter 2*a*_eff_ = 13.5 mm and apodization function *P* (*r*) = *P*_0_ |1−(*r*/*a*_eff_)^6^ | for the source pressure equal to 0.263 MPa, 0.37 MPa and 0.45 MPa (average acoustic power equal to 0.7 W, 1.4 W and 2.1 W), respectively.

**Figure 4:**
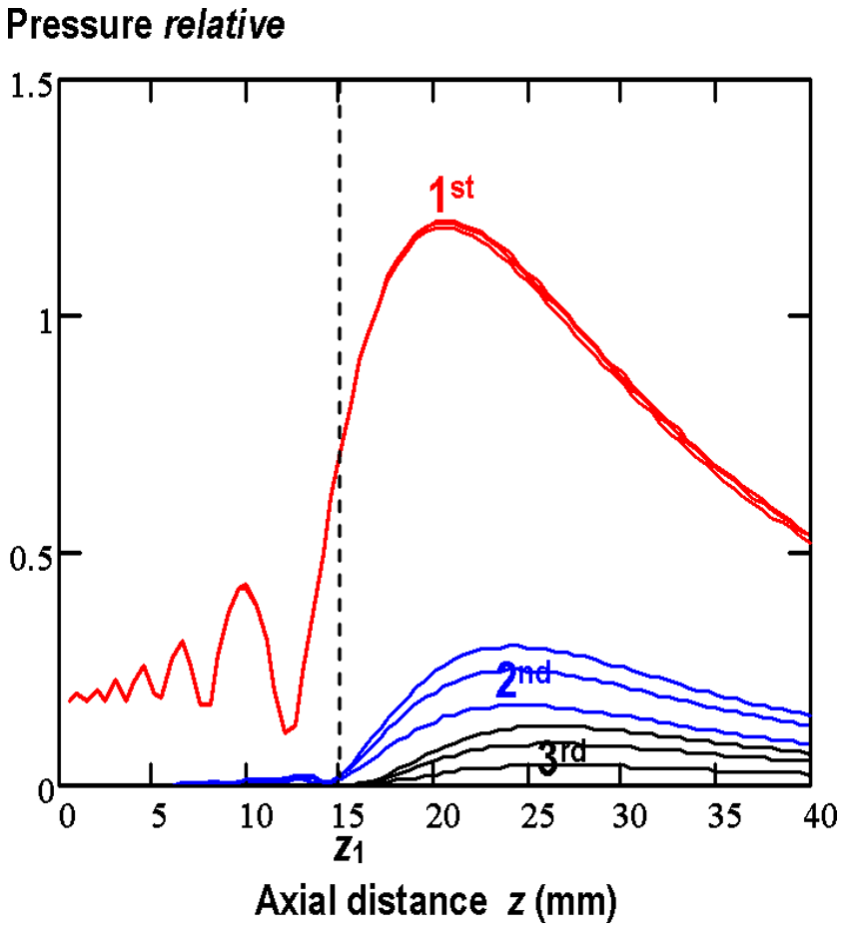
Calculated axial pressure variations of the 1^st^, 2^nd^ and 3^rd^ harmonic component for the pulsed nonlinear sound beam produced in water by 20-cycle tone bursts with a 0.2 duty-cycle and the initial acoustic pressure amplitude of 0.335 MPa, 0.475 MPa and 0.585 MPa.

In order to account for the effects of nonlinear propagation (generation of harmonic waves whose absorption increase nonlinearly with frequency) on temperature rise induced in tissue the distance between the transducer centre and water/tissue interface was determined (using the nonlinear propagation model in water) as the axial distance from the source at which the amplitude of the 2^nd^ harmonic component starts to be discernible. As was mentioned above this distance is specific for each circular source with *ka*<<1 (*k* is the wave number, *a* denotes the transducer radius) producing in water weak or moderate nonlinear sound field and is independent on the source pressure level. For the transducer used this distance was found to be 15 mm independently on the source pressure varied between 0.263 MPa and 0.45 MPa. These source pressures provided the weak or moderate nonlinear fields in water. Fig. 4 shows the axial pressure variations of the 1^st^, 2^nd^ and 3^rd^ harmonic component calculated for the 8-cycle finite-amplitude tone burst propagating in water.

### Methodology of Measurements

The beef liver *in vitro* was obtained from the Mokobody Company Ltd Poland (which deals in the purchase of live cattle, slaughter of animals and sale of meat and meat offal of bovine animals) and stored in a 1% saline solution. Experiments were done within 8 hours after slaughter. Before experiments each beef liver sample was degassed and inserted in a 30 mm diameter and 30 mm height cylindrical chamber. The chamber had an acoustically transparent, 20 µm thick Mylar film stretched over each end and was immersed in a water bath. The water/tissue interface was perpendicular to the acoustic beam axis and the separation distance between the interface and transducer centre was chosen to be 15 mm (the distance at which the second harmonics amplitude in the spectrum of the nonlinearly distorted pulse propagating in water starts to grow rapidly). The chamber remained in the water bath until the temperatures of water and beef liver equalized. Five thermocouples (with diameter of 0.2 mm) rigidly connected with each other were located in the beef liver sample through thin, 0.5 mm diameter hypodermic needles inserted through the holes in the water bath cover ensuring their precise position on the acoustic beam axis. The uncertainty of each thermocouple position was about ±0.5 mm. Due to small diameter of the thermocouples their influence on measurement results was negligible. The intervals between thermocouples were chosen to be 5 mm. The 1 mm distance between the holes in the water bath cover allowed to measure the temperature-time *T*(*t*) rises on the acoustic beam axis every 1 mm.

## Results

**Figure 5:**
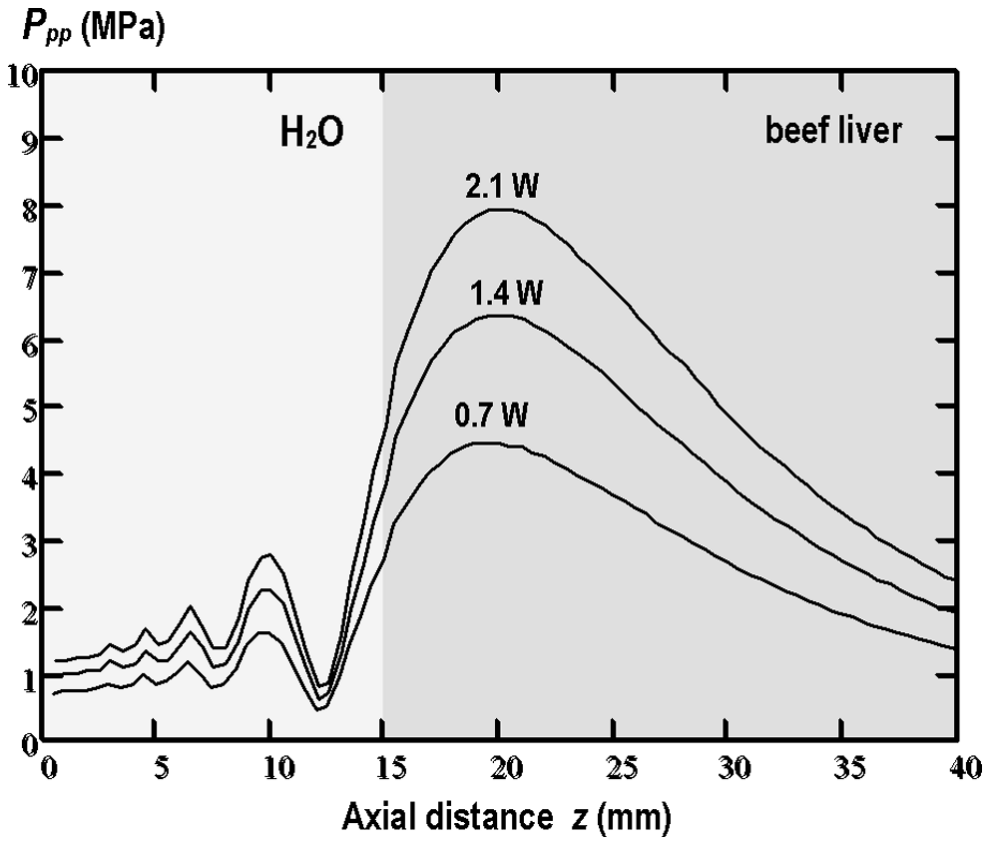
Axial peak-peak pressure variations in the pulsed focused nonlinear sound beam produced in the two-layer system of media (15 mm water/25 mm beef liver) by 20-cycle tone bursts with a 0.2 duty-cycle and calculated for the beams with 3 different average acoustic powers (0.7 W, 1.4 W and 2.1 W).

The tissue whose thermal conductivity was verified experimentally using the proposed method was the fresh beef liver *in vitro*. The acoustic and thermal parameters of water and beef liver required for the numerical model were available from literature and are quoted in Table 2 and Table 3. The temperature rises induced in beef liver during 10 min exposure to pulsed focused ultrasound with three different average acoustic powers varied between 0.7 W and 2.1 W were measured by thermocouples. The maximum heat position on the beam axis was determined for each acoustic power by comparison of the temperature-time *T*(*t*) plots registered by thermocouples. The measured axial position of the maximum heating spot was compared with the simulated one for each average acoustic power used (0.7 W, 1.4 W and 2.1 W). The measured and calculated position of the maximum heating was found to be at the same axial distance of 20 mm from the transducer centre as the position of the peak-peak pressure maximum in the nonlinear beam produced in the two-layer system of media used: water/beef liver (see Fig. 5).

**Figure 6:**
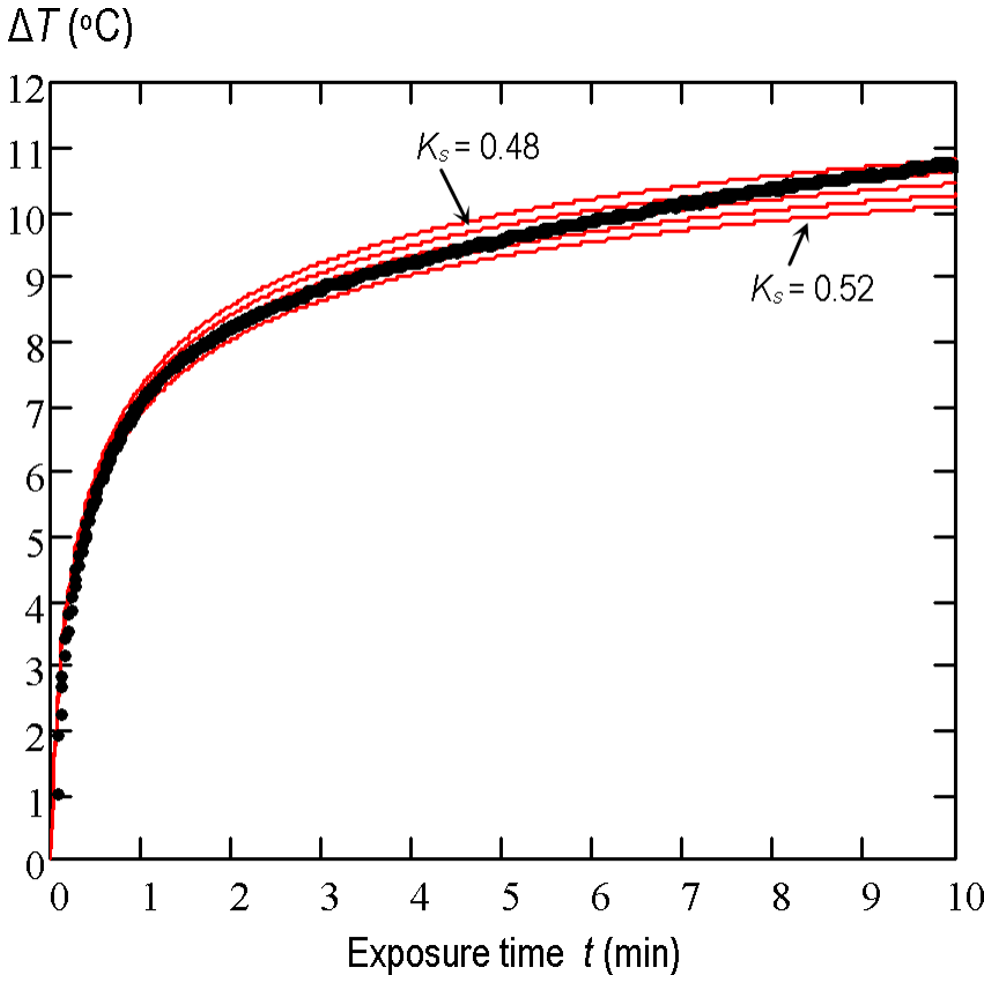
The temperature-time rise induced in the two-layer system of media. : 15 mm water/25 mm beef liver by the pulsed focused nonlinear beam with the average acoustic power of 0.7 W. The measurement data (points) and numerical simulation results (solid lines) at the point of maximum heating (*z* = 20 mm) for the assumed thermal conductivity of the beef liver varied between 0.48 W/(m·°C) and 0.52 W/(m·°C) with an increment of 0.01 W/(m·°C).

**Figure 7:**
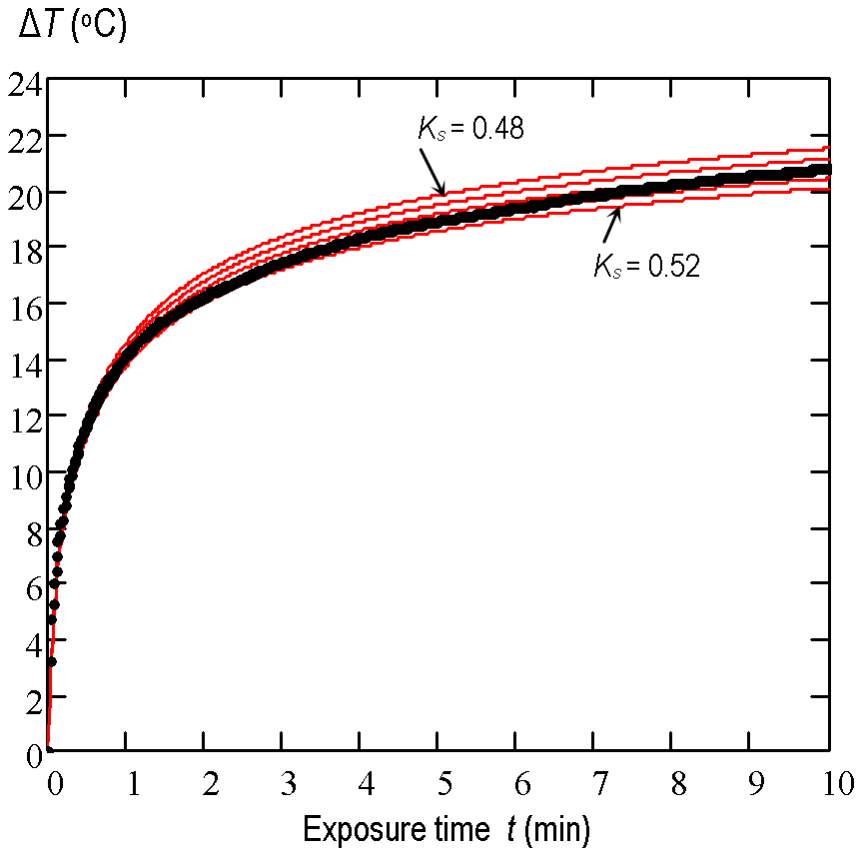
The temperature-time rise induced in the two-layer system of media. : 15 mm water/25 mm beef liver by the pulsed focused nonlinear beam with the average acoustic power of 1.4 W. The measurement data (points) and numerical simulation results (solid lines) at the point of maximum heating (*z* = 20 mm) for the assumed thermal conductivity of the beef liver varied between 0.48 W/(m·°C) and 0.52 W/(m·°C) with an increment of 0.01 W/(m·°C).

**Figure 8:**
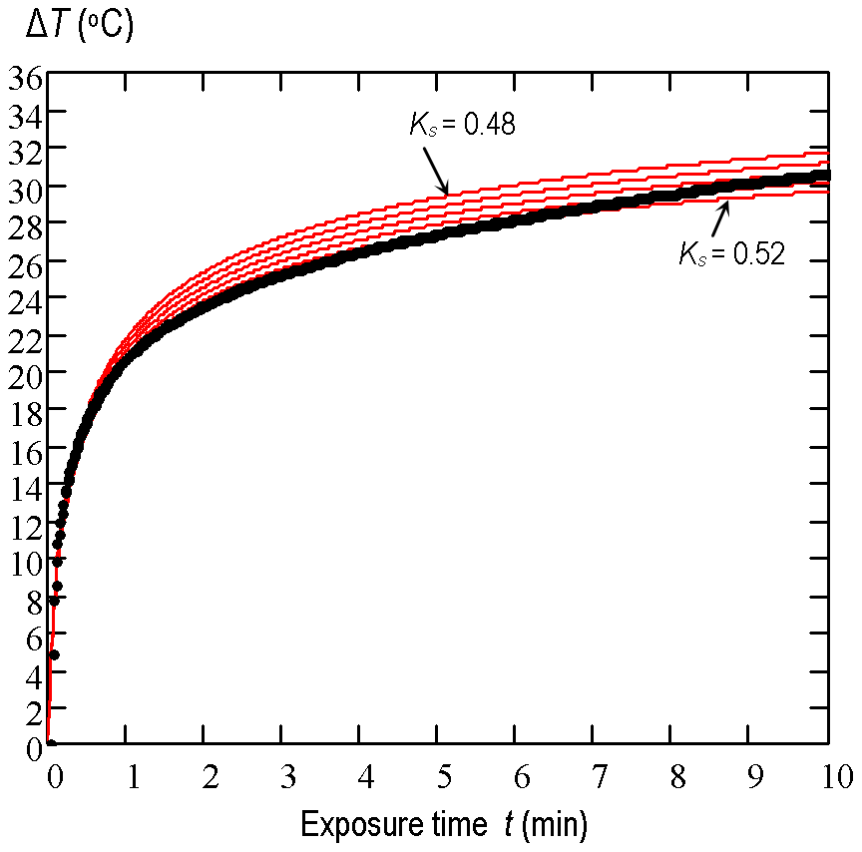
The temperature-time rise induced in the two-layer system of media. : 15 mm water/25 mm beef liver by the pulsed focused nonlinear beam with the average acoustic power of 2.1 W. The measurement data (points) and numerical simulation results (solid lines) at the point of maximum heating (*z* = 20 mm) for the assumed thermal conductivity of the beef liver varied between 0.48 W/(m·°C) and 0.52 W/(m·°C) with an increment of 0.01 W/(m·°C).

The measured temperature-time *T*(*t*) plot at the point of maximum heating was compared with the simulated one while thermal conductivity of beef liver was varied until the theoretical and experimental plots coincide as closely as possible. Simulations were made for the assumed thermal conductivity *K_s_* of beef liver varying between 0.48 W/(m·°C) and 0.52 W/(m·°C) with an increment of 0.01 W/(m·°C). The measured temperature-time *T*(*t*) rise and the set of the simulated plots for each acoustic power level used are shown in Fig. 6, Fig. 7 and Fig. 8. The best correlation coefficient (0.987) for the beam with the average acoustic power of 0.7 W was obtained when *K_s_* was equal to 0.49 W/(m·°C). For the 1.4 W and 2.1 W beams the best agreement between the theoretical results and experimental data was found to be for *K_s_* = 0.5 W/(m·°C) and *K_s_* = 0.52 W/(m·°C), respectively. The set of simulated curves shows that for the source boundary condition parameters considered here the temperature rise induced in beef liver *in vitro* at the point of maximum heating is directly proportional to the average acoustic power of the beam used or to the square of the source pressure amplitude.

The cause of the discrepancy between the measured and simulated results may be the accuracy of calibration of both, the hydrophones and thermocouples, the uncertainty in the frequency-dependent attenuation law assumed for beef liver, the uncertainty in the pressure distribution at the transducer surface, the uncertainty in the transmission properties of the Mylar film at higher temperatures as well as the tissue heterogeneity. Finally, the significant factor that may cause the discrepancy between measured and simulated results is the accuracy of positioning (especially in radial direction) of the thermocouple on the acoustic beam axis at the point of maximum heating.

## Discussion

The thermal conductivity *K_s_* determined for the beef liver *in vitro* by the proposed method is similar to the values used by other authors [13], [14]. The theoretical model used in this work is valid for determining the thermal conductivity of liquids and biological tissues whose attenuation law satisfy the equation *α* (*f*) = *α*_1_·*f ^b^*, where 1≤*b*≤2, and for which the nonlinear waveform distortion of the propagating plane finite-amplitude sound pressure wave can be described by the nonlinear wave equation.

The sensitivity of the proposed method depends on several factors such as the source frequency, diameter and f-number as well as the accuracy of the thermocouple positioning. Maximization of the transducer diameter and f-number is preferable for increasing the beam width in the focal plane. This helps to minimize the uncertainty of measurement of the temperature-time *T*(*t*) rise caused by the uncertainty of the thermocouple positioning in radial direction. In order to ensure accuracy of the nonlinearly distorted waveform measurements it is preferable to select the transducer with a low centre frequency because of limited range of the hydrophone's sensitivity characteristic (broadband bilaminar PVDF hydrophone used here was calibrated up to 40 MHz). To provide an accurate prediction of measured sound fields in water using the numerical model based on the transformed KZK equation the transducer excitation level should be selected so that the generated nonlinear field was weak or moderate. As already mentioned, this condition was determined in Nachef *et al.* showing that the ratio of the discontinuity distance to the Rayleigh distance for weak or moderate nonlinear fields should be larger than 0.3. Physically, this condition means that the shock forms in the transition- or far-field region of the acoustic beam. Calculation of the source pressure amplitude *P*_0_ making use of this inequality indicates that for the conditions considered here the source pressure has to stay above the linear mode amplitude but below 1.034 MPa. Indeed, this condition was fulfilled here - the source pressure amplitudes used were between 0.263 MPa and 0.45 MPa.

The obtained results (Fig. 6, Fig. 7 and Fig. 8) show that the temperature rise induced in beef liver samples during their exposure to pulsed focused nonlinear ultrasound beams with average acoustic power varied from 0.7 W to 2.1 W is directly proportional to the power value or to the square of the source pressure amplitude.

The accuracy of the developed method depends most of all on the positioning accuracy of the thermocouple in the beam focus (especially in radial direction) and on the accuracy of measurements of the source pressure amplitude required for the model as the input parameter. The error in the thermocouple positioning in radial direction within ±0.5 mm leads to 30% error in determination of thermal conductivity of the tested tissue. The variation of ±5% in determination of the initial pulse pressure amplitude leads to the variation of ±12.7% in evaluation of the thermal conductivity. Therefore, perfectly calibrated measuring hydrophones should be used. Another source of uncertainty is related to estimation of the power index *b* determining dependence of the tissue attenuation on frequency. The low accuracy of measurements of the tissue attenuation law over a broad frequency range may affect the accuracy of the temperature rise and impose limitations on the proposed method. For the most biological tissues *b* = 1∼1.3. For the value considered here (*b* = 1.1) variation of ±10% in this index leads to variation of about ±6.5% in evaluation of the beef liver thermal conductivity. Finally, the low accuracy in determination of the specific heat of the tested tissue does not really affect the accuracy in determination of the tissue thermal conductivity. Variation of ±10% in determination of the specific heat leads to variation of about ±1% in evaluation of the thermal conductivity using the proposed method.

In conclusion, the proposed method is relatively simple to use from the theoretical and practical point of view and is suitable for determining the thermal conductivity of some animal tissues *in vivo* (for example, a rat liver). It can be done by extension of the numerical model used (introducing the terms accounting for the effects of both, the blood perfusion losses and metabolic heat generation) and by location the thermocouple in the anesthetized rat liver in the acoustic beam focus (lying at a depth of few millimeters under the skin). The experimental determination of thermal conductivity of a rat liver i*n vivo* using the proposed method will be the next stage of our study.
